# Structure-Based Analysis of Five Novel Disease-Causing Mutations in 21-Hydroxylase-Deficient Patients

**DOI:** 10.1371/journal.pone.0015899

**Published:** 2011-01-11

**Authors:** Carolina Minutolo, Alejandro D. Nadra, Cecilia Fernández, Melisa Taboas, Noemí Buzzalino, Bárbara Casali, Susana Belli, Eduardo H. Charreau, Liliana Alba, Liliana Dain

**Affiliations:** 1 Centro Nacional de Genética Médica, ANLIS, Buenos Aires, Argentina; 2 Departamento de Fisiología Biología Molecular y Celular, Facultad de Ciencias Exactas y Naturales, Universidad de Buenos Aires, Buenos Aires, Argentina; 3 Departamento de Química Biológica, Facultad de Ciencias Exactas y Naturales, Universidad de Buenos Aires, Buenos Aires, Argentina; 4 Instituto de Biología y Medicina Experimental, CONICET, Buenos Aires, Argentina; 5 División Endocrinología, Hospital Durand, Buenos Aires, Argentina; University Paris 7 - Institut Pasteur, France

## Abstract

Congenital adrenal hyperplasia (CAH) due to 21-hydroxylase deficiency is the most frequent inborn error of metabolism, and accounts for 90–95% of CAH cases. The affected enzyme, P450C21, is encoded by the CYP21A2 gene, located together with a 98% nucleotide sequence identity CYP21A1P pseudogene, on chromosome 6p21.3. Even though most patients carry CYP21A1P-derived mutations, an increasing number of novel and rare mutations in disease causing alleles were found in the last years. In the present work, we describe five CYP21A2 novel mutations, p.R132C, p.149C, p.M283V, p.E431K and a frameshift g.2511_2512delGG, in four non-classical and one salt wasting patients from Argentina. All novel point mutations are located in CYP21 protein residues that are conserved throughout mammalian species, and none of them were found in control individuals. The putative pathogenic mechanisms of the novel variants were analyzed *in silico.* A three-dimensional CYP21 structure was generated by homology modeling and the protein design algorithm FoldX was used to calculate changes in stability of CYP21A2 protein. Our analysis revealed changes in protein stability or in the surface charge of the mutant enzymes, which could be related to the clinical manifestation found in patients.

## Introduction

Congenital adrenal hyperplasia (CAH) due to 21-hydroxylase deficiency (OMIM 201910) accounts for 90–95% of CAH cases [1], [2]. This autosomal recessive disorder, which is the most frequent inborn error of metabolism, has a broad spectrum of clinical forms, ranging from severe or classical, which includes the salt-wasting (SW) and simple virilising (SV) forms, to the mild late onset or non-classical form of CAH (NCCAH) [1]. Poor synthesis of cortisol, with or without aldosterone deficiency, results in chronic stimulation of the adrenal cortex by corticotropin (ACTH). Consequently, overproduction of some cortisol precursors are shunted into the androgen biosynthetic pathway, causing the signs and symptoms of androgen excess observed in this disorder. Girls with classical CAH are typically born with ambiguous genitalia due to the exposure to high systemic adrenal androgen levels from the 7th week of gestation. Patients with a concurrent defect in aldosterone biosynthesis also present life-threatening conditions which usually appear at 1–4 weeks of age in both sexes. The moderate enzyme deficiency that results in NCCAH is characterized by signs of hyperandrogenism such as early pubarche, hirsutism, oligomenorrhea or amenorrhea, polycystic ovaries, acne, and/or infertility.

Neonatal screening programs performed since 1977 have shown an overall incidence of 1∶15000 live births for the classical form [3]–[6]. A significantly higher prevalence for NCCAH (approximately 1∶1000 worldwide) have been estimated, being more frequent in certain ethnic groups such as Jews of Eastern Europe, Hispanics and Yugoslavs [7].

The affected enzyme, P450C21, is encoded by the CYP21A2 gene. It is located on chromosome 6p21.3 adjacent to a pseudogene, CYP21A1P, with which it shares 98% nucleotide sequence identity. Due to the high degree of identity between this gene and its pseudogene, most of the disease-causing mutations described are likely to be the consequence of non-homologous recombination or gene conversion events [8], [9]. In addition, about 130 rare point mutations that arise independently of the pseudogene and that were found specific to a population or a single family, have been described to date (for details: http://www.hgmd.cf.ac.uk).

In the present work, we describe five novel CYP21A2 mutations in patients from Argentina, diagnosed with 21-hydroxylase deficiency. In addition, using a three-dimensional molecular CYP21 model, the putative pathogenic mechanism of each mutation was evaluated *in silico*. Our analysis revealed changes in protein stability or in the surface charge of the mutant enzymes.

## Materials and Methods

All clinical investigations were conducted according to the principles expressed in the Declaration of Helsinki. Written informed consent was obtained from all patients and parents involved in this study. The study was approved by the Ethic Committee of the Instituto de Biología y Medicina Experimental, Buenos Aires, Argentina.

### Patients

Endocrine and genetic evaluation of patients were conducted at the Division Endocrinología of the Hospital Durand and at the Centro Nacional de Genética Médica, Buenos Aires, Argentina.

Patients were included following the diagnostic criteria already described [10], [11]. Briefly, a clinical diagnosis of NC 21-hydroxylase deficiency was established on the basis of late-onset symptoms of androgen excess, elevated basal 17-hydroxyprogesterone (17OH-P) (>2 ng/ml), and an abnormal -at least three-fold the upper limit of normal- 60 minute 17OH-P response to Synacten stimulation (>10 ng/ml). The SW form of the disease was characterized by elevated 17OH-P concentrations and plasma renin activity (>4,50 ng/ml/h), onset of hyperkalaemia, hyponatraemia, dehydration and/or shock in the first month of life, with the consequent requirement of mineralocorticoids and glucocorticoids treatment. Females presented with ambiguous genitalia. The SV form was diagnosed on the basis of elevated 17OH-P levels and ambiguous genitalia in females, and no evidence of salt wasting.

Details on clinical manifestations observed in patients in whom novel mutations were found, are presented in Supporting Information (Text S1).

### Hormone assays

17-hydroxyprogesterone (17OHP), androstenedione (A_4_), testosterone (T) and dehydroepiandrosterone-sulphate (DHEA-S) were assayed by radio-immunoassay (RIA) using a commercial kit from Diagnostic System Laboratory (DSL), Houston, TX, USA. Plasma Renin Activity (PRA) was measured using a commercial kit from Renin, BioChem Immuno System, Rome, Italy (MAIA®), as previously described [10], [11].

### DNA Analyses

Nucleotide numbering was performed following the guidelines of the Human Genome Variation Society [12], using M13936.1 [13] as the genomic CYP21A2 reference sequence. All new data has been deposited in the GeneBank database.

DNA was isolated from peripheral blood leucocytes and the 10 most frequent derived-pseudogene point mutations in the CYP21A2 gene were screened, following methodology previously established in our laboratory [10], [11]. Briefly, CYP21A2 gene was amplified by PCR in three overlapping fragments, and each mutation was screened by allele-specific PCR or PCR-RFLP in a second round of PCR using one of these previously amplified fragments as templates.

Samples from patients with at least one non-determined allele were further analyzed by direct sequencing. The entire coding and proximal promoter regions of the CYP21A2 gene were specifically amplified in four overlapping fragments using primers already described [14]–[16]. Fragment 1 included the promoter region up to exon 3, fragment 2 the region from exon 2 to exon 6, fragment 3 included the region from exon 3 to intron 7 and fragment 4 the region from exon 6 to exon 10. PCR products were purified by GFX PCR DNA and a Gel Band Purification Kit (GE Healthcare Bio-Sciences, Cardiff, Wales, UK), following the Big Dye terminator sequencing protocol (Applied Biosystems, Foster City, CA, USA). Fluorescent samples were analyzed using an ABI prism 3730XL DNA sequencer.

Each fragment was sequenced at least twice (forward and reverse orientation), and novel mutations were retested in at least two independent PCRs. In addition, each mutation was further analyzed by restriction enzyme digestion (see below). In order to establish the segregation of mutated alleles, DNA from parents was analyzed, when available. Alternatively, two independent allele-specific PCR fragments were amplified using primers encompassing the wild-type or the mutant p.V281L allele, in order to determine if the novel mutation was located in *trans* with the already identified mutation (patients 2 and 4). Each of these fragments was further analyzed by direct sequencing.

In addition, 50 randomly selected subjects from the general population were recruited and DNA samples were screened for the novel mutations found in the patients. Briefly, CYP21A2 was specifically amplified in two overlapping fragments (from the promoter region to exon 6 and from exon 3 to exon 10, respectively), and each mutation was further analyzed in a second round of PCR. Screening of the g.782C>T mutation in exon 3 was performed by amplification of a 696-bp fragment from exon 3 to exon 6 with primers already described [14], [15], followed by a BtsCI restriction enzyme assay and a 2% Ethidium Bromide-stained agarose gel electrophoresis. The wild type allele rendered fragments of 77, 200 and 419 bp, while the mutant alleles showed fragments of 200 and 496 bp. For the screening of mutations g.940C>T in exon 4, g.1695A>G in exon 7 and the g,2511_2512delGG in exon10, each exon was amplified with primers already described [16], and PCR products were analyzed by digestion with HhaI, Hin1II and BseDI restriction enzymes, respectively. PCR product from exon 4 was electrophoresed in 2% agarose gels stained with Ethidium bromide. The mutant allele rendered one uncut fragment of 355 bp, while the wild type allele rendered two fragments of 320 and 35 bp. Fragments from exon 7 and 10, were electrophoresed in 8% acrylamide-bisacrylamide gels and silver-stained. The 274-bp fragment from exon 7 rendered five fragments of 12, 27, 42, 72 and 121 bp in the wild-type alelle and four fragments of 12, 27, 72, and 163 bp in the mutant one. The 389-bp fragment from the wild-type alelle in exon 10 rendered fragments of 66, 77, 83 and 141 bp (along with fragments of low molecular weight), while in the mutant allele, fragments of 66, 83 and 218 bp were obtained. Mutation g.2515G>A [17] was screened using primer 10F [16] and a reverse mismatch one 5′CCAGGCGCGCCAACCGCT3′ (mismatch base underlined). The 166-bp fragment was digested with BsrbI restriction enzyme followed by 8% acrylamide-bisacrylamide gel electrophoresis and silver-stained. The mutant allele remained uncut, while the wild-type allele rendered two fragments of 150 and 16 bp.

Sequence analysis was performed using Blast-N algorithms. In addition, similarities between different mammalian CYP21 proteins were assessed using Uniprot reference sequences and the Clustal W multiple alignment program.

### Modeling novel mutations

Semi-automatic homology modeling was carried out with Biskit suite [18], which uses Modellerv91 as a modeling tool. Multiple templates, ranging from 4 to 18, were used to generate the different target models, having slightly better results using 18 templates. Table S1 presents all the templates used and its corresponding identity to CYP21 protein in the aligned region. The model that presented the best Modeller objective function value and DOPE score was selected to be used in the following analyses (model available upon request).

Once the best model was chosen, we used Foldx 3.0 beta (www.foldx.crg.es) to generate point mutations and analyze changes in protein stability. Since Foldx cannot deal with heme atoms, mutations in residues that directly interact with the prostetic group may be misleaded. Prior to any mutagenesis, the RepairPDB FoldX command was used to optimize the total energy of the protein. Mutagenesis was carried out using the BuildModel FoldX command, and each mutation was repeated five times for each structure. Protein stabilities were calculated using the Stability command, and ΔG values were estimated as the difference between the energy of the wild type protein and that of the mutant enzyme. A threshold of 1,6 kcal/mol was considered, as it corresponds to twice the standard deviation calculated with FoldX and values above this threshold should significantly destabilize a protein [19].

The five novel point mutations found in patients from our cohort, together with 40 already described point mutations, were also tested using a previously described 3D model (Protein Data Bank ID:2GEG, [20]). Each mutation was performed five times with both templates and the results were expressed as mean plus standard deviations.

## Results

**Figure 1:**
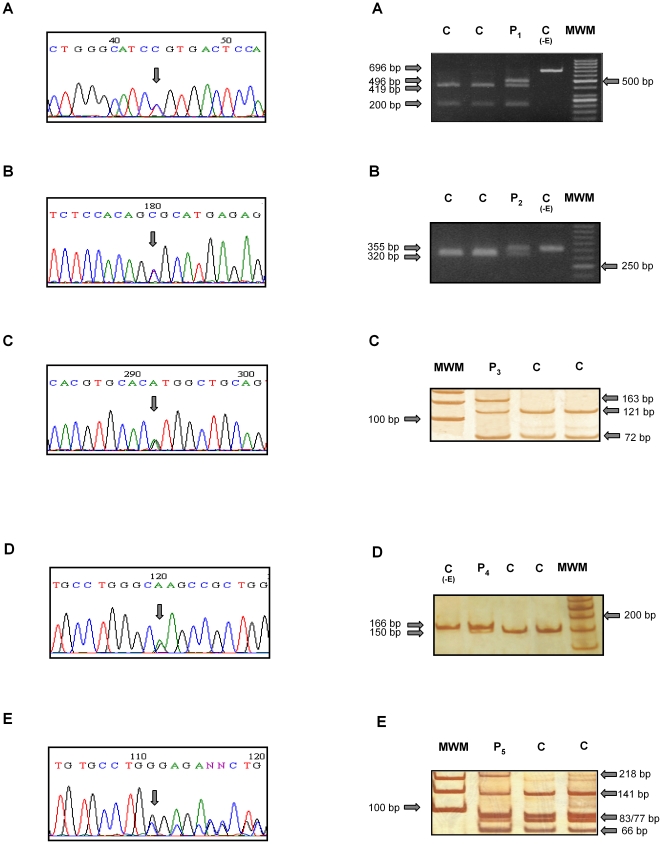
Representative electropherograms (left panel) and restriction enzyme assays (right panel) of the five novel mutations found. Left panel: **A**: Mutation g. 782C>T, in exon 3, changes an R residue to C at position 132. **B**: Mutation g.940C>T, in exon 4, changes an R residue in position 149 to a C residue. **C**. Mutation g.1695A>G, in exon 7, predicts a change in residue M283 to V283. **D**: mutation g.2515G>A, in exon 10, changes E431 to K431. **E:** mutation g.2511_2512delGG in exon 10 leads to a frameshift in the carboxy-terminal end of the protein. Right panel: **A:** BtsCI restriction enzyme assay for R132C. **B**: HhaI restriction enzyme assay for R149C. **C**: Hin1II restriction enzyme assay for M283V. **D**: BseDI restriction enzyme assay for g.2511_2512delGG in exon 10. **E:** BsrbI restriction enzyme assay for E431K. C: control individuals, P: patients. C(-E): control without enzyme. MWM: molecular weight marker.

Following an initial screening of the 10 most frequent pseudogene-derived mutations, direct sequencing of the complete coding and the proximal promoter regions was performed in those patients with at least one non-determined allele. This combined strategy revealed the presence of five novel mutations in four NCCAH and one SW 21-hydroxylase-deficient patients from our cohort. Representative electropherograms and restriction enzyme digestion assays for the novel mutations are shown in Figure 1. Table 1 summarizes the phenotypes, genotypes and biochemical parameters of patients in whom new variants were found. None of these mutations were found in 100 chromosomes of randomly selected individuals from the general population.

**Table 1:** Phenotype, genotype and hormonal values in the five patients in whom novel mutations were found.

Patient	Phenotype	Gender	Genotype	17-OHP (basal)	17-OHP (stimulated)	Δ_4_A	To	DHEA-S
1	NC	F	**p.R132C**/N	5.7	30	ND	0.6	3430
2	NC	F	p.V281L/**p.R149C**	8.5	34	5.2	0.55	2860
3	NC	F	IV2-13 A/C>G/**p.M283V** a	19	ND	6.3	1.4	1500
4	NC	F	p.V281L/p.D322G-**p.E431K**	23	ND	4.8	1.2	1800
5*	SW	F	**g.2511_2512delGG**/p.I172N^b^	10	ND	12.4	1.64	1802

Novel mutations are displayed in bold and hormonal values are expressed in ng/ml. Values of basal 17 hydroxyprogesterone (17-OHP) and after ACTH stimulation, basal androstenedione (Δ_4_A), testosterone (To) and dehydroepiandrosterone sulphate (DHEA-S) are showed. *Hormonal values under treatment with 6 mg meprednisone plus 0.05 mg 9α-fluorocortisol.

a: cis/trans presence of mutations is assumed.

b**:** maternal/paternal alleles.

**ND**: not determined. **N**: Normal wild type allele.

**Figure 2:**
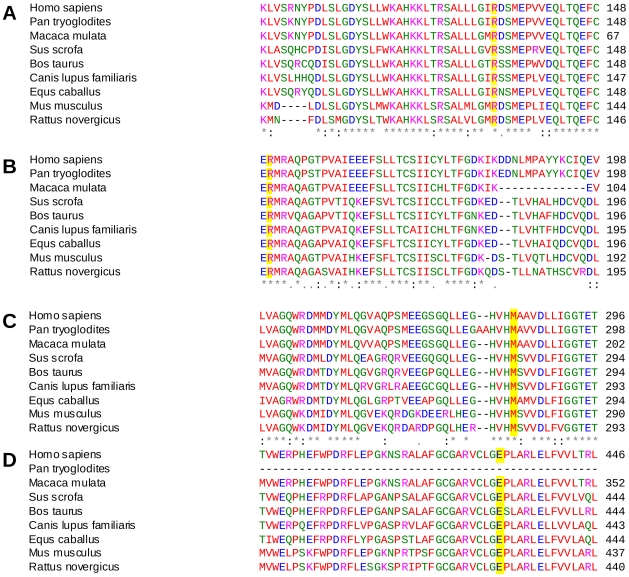
Partial Clustal W alignment analyses of CYP21A2 proteins from different mammalian species. The residues of each mutation are highlighted in yellow. **A:** R residue at position 132. **B**: R residue at position 149. **C**. M residue at position 283. **D:** E residue at position 431.

In patient 1, due to the presence of g.782C>T mutation in exon 3, an arginine residue is replaced by a cysteine at position 132**.** In patient 2, g.940C>T mutation located in exon 4 replaces an arginine residue with a cysteine at position 149, and in patient 3, g.1695A>G mutation in exon 7 leads to a change in residue M283 to V283. In addition, while g.2515G>A mutation in exon 10 evidenced in patient 4 replaces an aspartic acid with a lysine at position 431, g.2511_2512delGG found in patient 5, leads to a frameshift in the carboxy-terminal end of the protein. As shown in Figure 2, all novel point mutations are located in CYP21 protein residues that are conserved throughout mammalian species.

**Figure 3:**
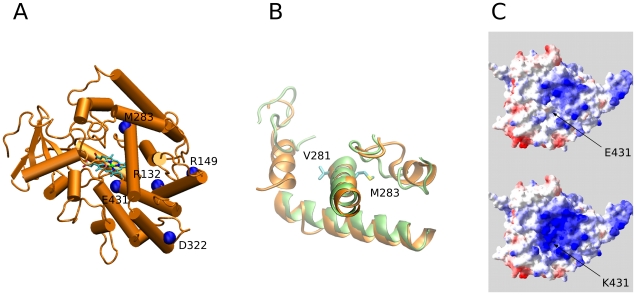
Structural analysis. **A**: Cartoon representation of the structure of CYP21A2. Residues implicated in the novel mutations found are labeled and highlighted by blue spheres. The residue D322 is also represented. Heme cofactor is depicted in sticks. **B**: Model discrepancies in the surroundings of V281. Detail of the superimposition of the Biskit (green) and 2GEG (orange) models. V281 and M283 are named and depicted in sticks. **C**: Enhancement of the basic region in the mutant surface. Surface electrostatics of the wild type E431 and mutant K431 are presented. Acidic regions are depicted in red and basic ones in blue.

The putative pathogenic mechanisms of the novel variants were analyzed *in silico* by a three-dimensional CYP21 structure generated by homology modeling. Figure 3A shows a cartoon scheme of the protein, highlighting the positions involved in point mutations. In this work we used a structure-based approach and the protein design algorithm FoldX to analyze these mutations and to calculate changes in stability of CYP21A2 protein. Table 2 summarizes the results obtained for the predicted free energy changes after amino acid substitution, using our model (Biskit, [18]) and a previously described one (2GEG [20]). For comparison, 40 mutations in which *in vitro* activities have been already assayed were also analyzed (see Supporting Information: Text S2, Table S2, Figure S1, Figure S2).

**Table 2:** Stability effects for the different point mutations found.

	Biskit	2GEG
**p.R132C**	0.1±0.06	4.13±0.08
**p.R149C**	1.88±0.18	1.68±0.32
**p.M283V**	−3.77±0.40	1.69±0.05
**p.E431K**	−0.20±0.17	−0.37±0.10

Changes in ΔG caused by mutations were evaluated by FoldX for our model (Biskit) as well as for a previously described one (2GEG). Values are expressed as kcal mol^-1^ with their corresponding standard deviation.

**p.R132C** (g.782C>T) mutation is located in the loop connecting helixes C and D, and lies in a region involved in the redox partner interaction [20]. Although this region is quite different in both models (Figure S3), in both cases p.R132C mutant disrupts favorable interactions between arginine and other residues (R254, A126 and L127 for 2GEG model and P432 backbone for Biskit, data not shown). Since R132 is far from the heme group and from other cysteine residues, no intra-protein bond is expected for the C132 residue.

**p.R149C** (g.940C>T) mutation is predicted to destabilize the protein in both models. C149 is close to C147, both are structured on the same D alpha helix and oriented in opposite directions (Figure S4). Consequently, no disulfide bridge is expected to be formed. Since this position is partially exposed to the solvent and away from the heme group, the putative effect on enzyme activity could be related to protein destabilization.

**p**.**M283V** (g1695A>G) is very close to p.V281L mutation (Figure 3B) in helix I, and it is also away from the heme group. In contrast to p.V281L (Table S2), while our model predicts that this mutation may stabilize the protein, a destabilization can be expected by 2GEG model. These discordances could be related to the differences found between both models in the predicted structure for this region of the protein (Figure S3).

**p.E431K** (g.2515G>A) mutation lies at the beginning of L helix and no protein destabilization is predicted (it marginally stabilizes the protein by ∼0.2 kcal/mol). Nevertheless, analyzing the structure in detail, this residue is found exposed, and the mutation involves a charge inversion from a negatively charged glutamic acid to positively charged lysine. As shown in Figure 3C, the p.E431K mutation significantly modifies the surface of the protein, potentially affecting its interaction with ligands. Based on molecular modeling, the effect of this mutation over protein stability is probably independent of p.D322G mutation found on the same allele. Both mutations are far away each other in the protein structure and are oriented in opposite directions (Figure 3A).

The frameshift **g.2511_2512delGG** mutation in exon 10 implies a huge change in the carboxy-terminal of the protein, therefore it cannot be accurately modeled with the present approach. Given that this mutation is located only one codon apart from the heme-coordinating cysteine, introducing 26 aminoacids and leading to a complete different tract of 91 residues, a non-functional protein lacking the heme prostetic group could be expected.

## Discussion

The adrenocortical 21-hydroxylase is one of the key enzymes in glucocorticoid and mineralocorticoid biosynthesis. Mutations in the CYP21A2 gene have been reported in individuals affected with CAH due to 21-hydroxylase deficiency. To date, a great number of different mutations in the CYP21A2 gene have been described. Most patients are compound heterozygotes, and their phenotype depends on the underlying combination of mutations they present [21]. Even though most patients carry CYP21A1P-derived mutations, an increasing number of naturally occurring mutations have been found in disease-causing alleles in the last years (see: http://www.hgmd.cf.ac.uk for details). Most of them result in amino acid substitutions that may disturb essential functional and/or structural motifs of the protein.

In recent years, efforts have been made towards predicting activities of mutant proteins. Given the practical impossibility to solve every mutant structure, and in order to avoid expensive and time-consuming *in vitro* activity assays, homology modeling emerges as a useful tool to evaluate, through structure-based methods, protein activity or stability impairment. Though this approach is being largely developed, it requires a high quality template in order to be reliable.

Given an adequate structure template, the FoldX algorithm allows the prediction of an eventual protein destabilization that, at the same time, can be correlated to a decrease in protein activity. When the mutation affects the active site, more sophisticated and time-consuming Quantum Mechanical computational techniques should be used. Structure-based energetic analysis has been successfully applied to the study of phenylketonuria [22], where a very good correlation between predicted and experimental protein stability – key factor in protein function- has been obtained.

In the present work, we describe five novel mutations found in 21-hydroxylase-deficient patients from our cohort. Considering that none of these mutations were found in the control individuals analyzed, and given the fact that all five mutations are located in CYP21 protein residues fully conserved throughout mammalian species, an impairment of 21-hydroxylase enzyme activity may consequently be suggested.

The putative pathogenic mechanisms of these newly described mutations were analyzed *in silico,* by means of mutagenesis modeling and protein stability calculations using our own generated model, as well as a previously available one. It should be noted that the quality and accuracy of predictions using the FoldX force field crucially depend on the availability of high resolution structures, usually determined by X-ray crystallography. In the case of P450CYP21A2, we are dealing with two main limitations: the lack of a high resolution protein structure, and the impossibility to evaluate the energetic of mutations affecting heme-interacting residues. Nevertheless, the use of approximate models allows to make some predictions with certain accuracy, and the development of a pipeline to be applied when the structure of the CYP21 protein becomes available.

In view of previous observations that demonstrated a correlation between the predicted protein stability and the severity of CAH [20], in the present work we also analyzed mutations with known *in vitro* enzymatic activity and compared our results with those obtained with the model proposed by Robins *et al* to further validate our findings. Considering only those regions that are well-conserved and similarly structured by both models, it is expected that in one third of the protein (mostly in the alpha helix) mutations could be evaluated accurately. An approximate evaluation can be carried out in another third of the protein. Little can be inferred about the remaining third, which is composed of heme-interacting residues and regions with diverging conformations between different models, thus suggesting that predictions in these regions of the protein may not be reliable. Indeed, we found a good correlation between experimental activity and predicted free energy when those mutants not likely to be involved in interaction with ligands were analyzed. In view of the different techniques employed to obtain both models, this convergence in the results may suggest that predictions for mutations in these regions of the protein may be consistent.

It is important to note that protein stability but not protein activity can be evaluated with this approach. In general, it is expected that a decrease in stability will affect activity; nevertheless, the opposite scenario may not be true. Such is the case of p.V281L mutation, for example, for which although both models predicted a stabilizing energy, previous *in vitro* analyses had demonstrated a decreased enzymatic activity [23], [24]. It should be highlighted, however, that in some cases where no stability impairment is found, the disease-causing effect could be explained by amino acid residues affecting other functions, e.g. heme coordination, post-translational modifications motif, interface with other interacting proteins or ligands.

After the initial analysis of mutations with known *in vitro* enzymatic activity, we further analyzed the novel mutations herein described using both models in parallel. Even though the region surrounding p.R132C mutation appears quite different in one model as compared to the other one, in both cases the C132 mutant disrupts favorable interactions. R132 is part of a cluster of basic amino acids suggested to be involved in the redox partner interaction with P_450_ Oxido Reductase (POR) [20]. Since mutations in these basic amino acids are likely to cause electrostatic disturbances, an impairment of the enzyme biological activity by C132 mutation would be expected. Nevertheless, the magnitude of this impairment can not be predicted. Robins et al. [20] developed a model used to describe single missense mutations and their predictive activities (http://bioinfo6.limbo.ifm.liu.se/cyp21/mutations.html). Nevertheless, the rough prediction proposed could represent an oversimplified outcome since it is based on a low-identity homology model. When dealing with proteins with no high resolution structure available, a trained eye may be necessary in order to draw meaningful conclusions. This is particularly important in cases in which multiple mutations are present and a cooperative effect needs to be evaluated.

It should be noted that under some circumstances the nature of the mutation present on the homologous allele together with the phenotype of the patient could contribute to predict the residual enzymatic activity disclosed by the novel mutation. However, in the case of patient 1, this putative assumption is not possible due to the lack of demonstrable mutation on the second allele. Though the entire gene and promoter regions were sequenced, no additional mutations were found. No variations were either found when we analyzed, by direct sequencing, two previously described regulatory regions [25], [26] (data not shown). The absence of mutations in patients diagnosed with 21-hydroxylase deficiency has been described previously [27]–[33] **.**

R149 residue, which lies in the D helix, may not be involved in known interactions with other proteins or cofactors. When modeling the protein with the C149 residue, no apparent partner to form a disulfide bridge can be predicted. Given the fact that both models suggest a significant destabilization for pR149C, we proposed that a change in the stability of the protein may be responsible for protein missfunction.

Residue M283 is located in the well-conserved helix I, which is suggested to be involved both in heme binding and in substrate recognition [20]. As we have already pointed out, both models disclosed different results for p.M283V mutation. While the 2GEG model showed no differences between the mutant and the wild-type protein in the free energy elicited, a huge difference in protein stability is predicted in our model. These discordances could suggest that none of the models may actually represent a good structural template for this particular region. Due to the proximity to p.V281L, the putative pathogenic mechanism of p.M283V mutation should be similar to that previously proposed for the former, as the impaired enzyme activity could be related to alterations in ligand recognition/migration or in protein-protein interactions [21]. Considering the *in vitro* activity of 281L, close to 30-50% [23], [24], and the almost null residual enzymatic activity of the protein codified by the homologous allele in this patient, we suggest that 283V may be associated with a mild phenotype. Interestingly, another point mutation at the same position, 283L, has previously been associated with the NC form of the disease [31].

Both models predicted no destabilization of the protein for the p.E431K mutation. It should be noted, however, that E431 lies within a cluster of basic residues located on the surface of the redox partner binding site of CYP21 [34]. Therefore, changes in the electrostatic potential in this surface, in our case adding an additional basic amino acid residue instead of an acidic one, could contribute to a miss-interaction with POR. Replacement of a glutamic acid by a basic lysine may affect the correct orientation of the ligands by disrupting the surface charge pattern or by enhancing the interaction energy in an already highly basic-residue dense region, thus potentially affecting enzyme dissociation.

p.E431K mutation was found to be located in *cis* with a previously described p.D322G (g.2012A>G) mutation [35]. Since functional *in vitro* studies have demonstrated that p.D322G impairs the activity of the enzyme [36], we cannot rule out that the pathological consequence for this allele might indeed rely on that mutation. Nevertheless, a significant difference in the final enzymatic activity has been reported when more than one mild mutation lies on the same allele [37]–[40]. Given the fact that the molecular modeling herein described revealed probably independent effects of these mutations over protein stability, i*n vitro* analyses seem necessary to further clarify the severity of this allele, for future appropriate genetic counseling.

Presence of the g.2511_2512delGG frameshift mutation in patient 5 introduces a complete new carboxy-terminal sequence to the protein. Although the protein is translated accurately up to the heme-coordinating cysteine at position 428, the new protein domain probably prevents the correct interaction with the heme group, thus rendering the protein enzymatically inactive. Notably, while the presence of the p.I172N mutation on the homologous allele may predict a SV form of the disease, the phenotype of the patient herein reported is consistent with a SW form of the disease. Similarly to patient 1, after analyzing the entire gene, promoter and regulatory regions, no additional mutations were found that could explain the lack of phenotype/genotype correlation. A frameshift mutation at the carboxy-terminal domain of the protein in nucleotide 2672 has been previously described [32], [41]. This frameshift mutation also predicted a new amino acid tract of 45 residues, but no phenotype/genotype discrepancy was found for the SV patient reported [32]. Considering that it has been suggested that the enzymatic residual activity of the p.I172N mutation is not always enough to prevent salt wasting [27], these observations as a whole might imply that other genetic and/or environmental factors may contribute to modulate the activity of the enzyme *in vivo.* In addition, the possibility that the newly generated carboxi-terminal domain could in fact act as a negative dominant protein should also be considered. It might be hypothesized that the mutated protein could be folded in a way which may allow it to quench natural ligands and/or to disrupt protein networks inside the cell, dramatically impairing the function of the protein synthesized by the homologous allele. In the near future, *in vitro* studies should be conducted -including co-expression with different mutant alleles- to further analyze the biological implication of the g.2511_2512delGG mutation.

In conclusion, we herein describe five novel mutations found in 21-hydroxylase-deficient patients from the Argentinean population. All novel mutations are located in CYP21 protein residues fully conserved throughout mammalian species. In all cases, molecular modeling revealed changes in the stability and/or surface charge of the protein that could be related to the clinical manifestation found in patients.

## Supporting Information

Text S1 **Clinical characteristics of patients in whom the novel mutations were found.**
(DOC)

Text S2 ***In silico*** **analysis of known P450CYP21A2 mutations.**
(DOC)

Figure S1 **Correlation between experimental activities and predicted stabilities**. Forty mutants in Human P450CYP21A2 protein with published *in vitro* functional studies were analyzed using an available theoretical model (2GEG) and our own generated three-dimensional structural CYP21 one (Biskit). The logarithm of the residual enzymatic activity on 17OH-P as a substrate was plotted against the predicted free energy change upon mutation. To aid the graphical representation, 0.1% activity was assigned in cases where 0% activity was reported.
(PDF)

Figure S2 **Correlation between experimental activities and predicted stabilities for both models.** Residues known to impair protein function independently of protein stability were excluded. Mutants with similar predicted stabilities (within 1 kcal mol^−1^), and thus more reliable, are depicted in open squares while those with different values, are in black triangles. In solid black, a trend-line considering all depicted values.
(PDF)

Figure S3 **Superimposition of models 2GEG (orange) and Biskit (green).** For clarity, only one heme is depicted in sticks. Both models tend to be more similar in regions with secondary structure. Residues implicated in the novel mutations found, as well as D322 residue are labeled
(PDF)

Figure S4 **Cartoon representation of the helix D.** Residues 147 and 149 (in sticks) point toward opposite directions.
(PDF)

Table S1 **Identity of the target sequence to the each template structure.** PDB codes and chain identifier for the 18 template structures used along with their sequence identity to the aligned region.
(DOC)

Table S2 **Predicted ΔG and** ***in vitro*** **enzymatic activity of 40 point mutations of human P450CYP21A2.** The mutations were analyzed *in silico,* by means of mutagenesis modeling and stability calculations using a previously available model and our own generated one, with the protein design algorithm FoldX.
(DOC)
