# Supplementary material for: Structure-Based Analysis of Five Novel Disease-Causing Mutations in 21-Hydroxylase-Deficient Patients
Source: PLoS One. 2011 Jan 11;6(1):e15899. doi: 10.1371/journal.pone.0015899 (PMC3019215; doi:10.1371/journal.pone.0015899)
Supplement: Text S1 — Clinical characteristics of patients in whom the novel mutations were found. (DOC) [file pone.0015899.s001.doc]

**Patient 1**: A twenty-six-year-old woman was referred to the Centro Nacional de Genética Médica with a presumptive diagnosis of Nonclassical Congenital Adrenal Hyperplasia (NCCAH) on the basis of hormonal assays.

She is the second child born from healthy non-consanguineous parents. She had menarche at the age of 13. She presented hirsutism and oligomenorrhea and was medicated with oral contraceptives since age 15. At the moment of our first interview, she was receiving 2mg/day of dexametasone.

**Patient 2:** A twenty-one-year-old woman was referred to the División Endocrinología of the Hospital Durand complaining of hirsutism since puberty. Her menarche occurred at age 11 and she presented regular menses since then. The hirsutism score, obtained by the Ferriman-Gallway method, was 13. She became spontaneously pregnant at 18 years of age.

**Patient 3:** A twenty-seven-year-old woman was referred to the División Endocrinología of the Hospital Durand complaining of moderate hirsutism since 17 years of age. The menarche occurred at the age of 12 and menstrual cycles were regular since then. The hirsutism score obtained by the Ferriman-Gallway method was 13. She was treated with 2mg meprednisone and a decrease in hair growth was observed.

**Patient 4:** A twenty-year-old woman who presented herself at the División Endocrinología of the Hospital Durand complaining of moderate hirsutism since the age of 15. Her menarche occurred at age 12, with irregular menses up to age 17. She presented moderate acne in thorax and facial hirsutism with a score of 12 (Ferriman-Gallway method). She was treated with 0.5 mg dexamethasone oral contraceptive (Diane 35®) and electrolysis therapy. At 35 years of age she stopped taking oral contraceptives and became pregnant.

**Patient 5:** A twenty-one-year-old woman was referred from the pediatric unit for ongoing management of congenital adrenal hyperplasia with salt-wasting due to 21-hydroxylase deficiency. She was diagnosed at birth due to ambiguous genitalia, including clitoromegaly and a single urogenital orifice, fusion of the labioscrotal folds, and poor weight gain. Pregnanetriol excretion was highly increased for her age. She was treated with oral hydrocortisone and 9α-fluocortisol since then. She had menarche at age 13, though she presented periods of oligomenorrhea and amenorrhea since then. The treatment compliance was adequate and the final height was 1.71m. When transferred to adult unit, treatment diagram was switched to 6mg meprednisone plus 0.05 mg 9α-fluorocortisol. Contraceptive pills were indicated when she reached 21.
