# Supplementary material for: Structure-Based Analysis of Five Novel Disease-Causing Mutations in 21-Hydroxylase-Deficient Patients
Source: PLoS One. 2011 Jan 11;6(1):e15899. doi: 10.1371/journal.pone.0015899 (PMC3019215; doi:10.1371/journal.pone.0015899)
Supplement: Text S2 — In silico analysis of known P450CYP21A2 mutations. (DOC) [file pone.0015899.s002.doc]

The putative pathogenic mechanisms of forty mutations in human P450CYP21A2 protein with already published *in vitro* functional studies were analyzed *in silico,* using our own generated model (Biskit [1]), as well as a previously available one (2GEG, [2]). In this work we subsequently used the protein design algorithm FoldX (www.foldx.crg.es) to analyze these mutations and to calculate changes in stability of CYP21A2 protein (Table S2). Figure S1 shows a cartoon depicting lack of correlation when the logarithm of the residual enzymatic activity, evaluated using 17OH-P as substrate, was plotted against the predicted free energy change upon mutation. Given that several mutations were reported to impair the enzymatic activity independently of its effects on protein destabilization, a detailed analysis was performed in those 25 mutations that were described to be not involved in interaction with ligands (Figure S2). In 18 mutations, similar energy values were predicted when analyzed either with one model or with the other one (squares in Figure S2). In view of the different techniques employed to obtain each model, this convergence in the results may suggest that predictions for mutations in these regions of the protein may indeed be reliable.

The R^2^ value obtained from the correlation between the logarithm of the experimental activity and the predicted free energy change -excluding those mutations known to interact with heme, ligand or other proteins- was 0.42 for Biskit model and 0.40 for 2GEG model (Figure S2). Nevertheless, if mutations p.I236N and p.P30Q are excluded from this analysis, the R^2^ rises to 0.63 and 0.53, respectively, being these last suitable values for a molecular modeling approach. p.I236N change lies in an structured alpha helix and is not proposed to be involved in heme or ligand binding. However, it was reported to have a strong effect impairing protein synthesis by an unknown mechanism [3]. On the other hand, p.P30Q mutation lies in a region without predicted secondary structure where both models may fail to be reliable enough.

# REFERENCES

1. Grünberg R, Nilges M and Leckner J. (2007) [Biskit - A software platform for structural bioinformatics.](http://biskit.pasteur.fr/publications/bioinformatics2007) Bioinformatics 23: 769-70
2. Robins T, Carlsson J, Sunnerhagen M, Wedell A, Persson B (2006) Molecular Model of Human CYP21 Based on Mammalian CYP2C5: Structural Features Correlate with Clinical Severity of Mutations Causing Congenital Adrenal Hyperplasia. Mol Endocrinol. 20:2946–2964.
3. Robins T, [Barbaro M](http://www.ncbi.nlm.nih.gov/pubmed?term="Barbaro M"%5BAuthor%5D), [Lajic S](http://www.ncbi.nlm.nih.gov/pubmed?term="Lajic S"%5BAuthor%5D), [Wedell A](http://www.ncbi.nlm.nih.gov/pubmed?term="Wedell A"%5BAuthor%5D) (2005) Not all amino acid substitutions of the common cluster E6 mutation in CYP21 cause congenital adrenal hyperplasia. J Clin Endocrinol Metab 90:2148-2153.
