# Supplementary figures and images for: Structure-Based Analysis of Five Novel Disease-Causing Mutations in 21-Hydroxylase-Deficient Patients
Source: PLoS One. 2011 Jan 11;6(1):e15899. doi: 10.1371/journal.pone.0015899 (PMC3019215; doi:10.1371/journal.pone.0015899)

# Figure S1

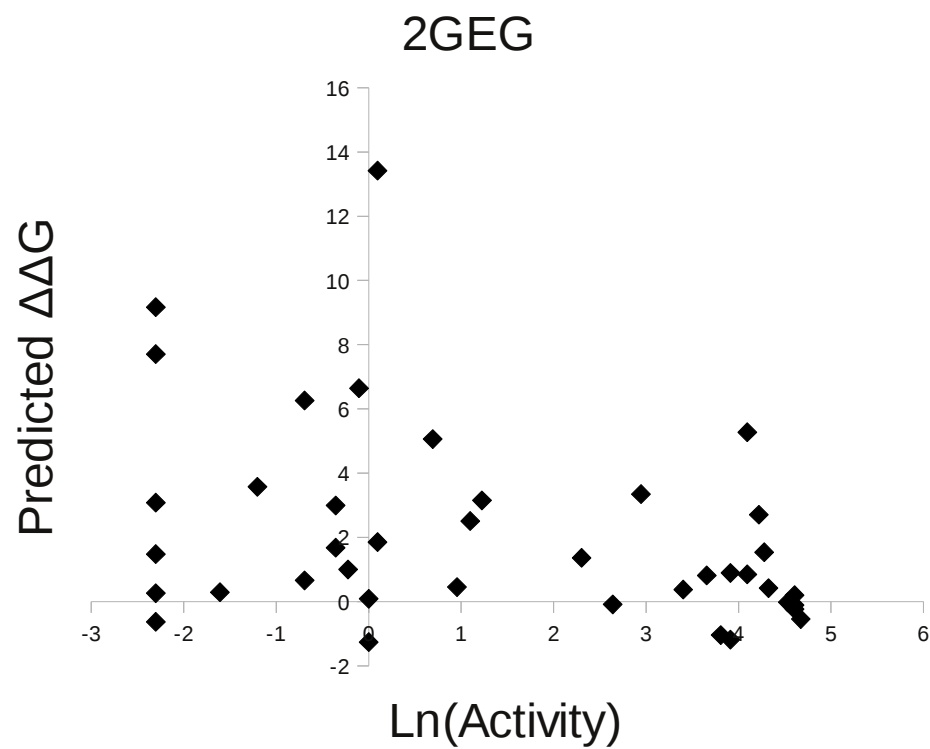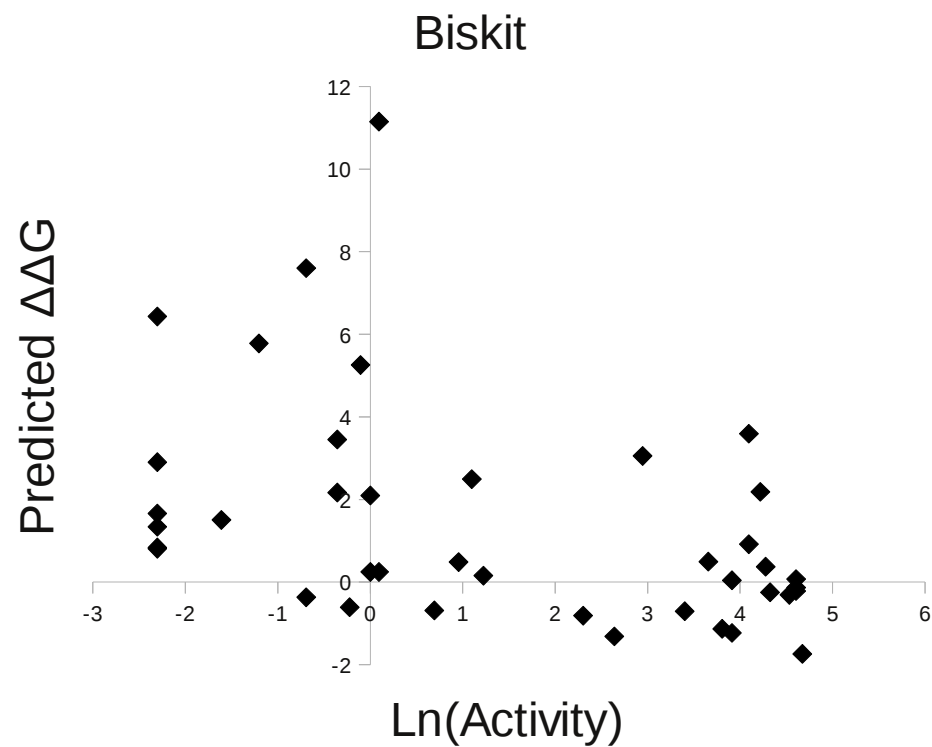

Supplement: Figure S1 — Correlation between experimental activities and predicted stabilities. Forty mutants in Human P450CYP21A2 protein with published in vitro functional studies were analyzed using an available theoretical model (2GEG) and our own generated three-dimensional structural CYP21 one (Biskit). The logarithm of the residual enzymatic activity on 17OH-P as a substrate was plotted against the predicted free energy change upon mutation. To aid the graphical representation, 0.1% activity was assigned in cases where 0% activity was reported. (PDF) [file pone.0015899.s003.pdf]

# Figure S2

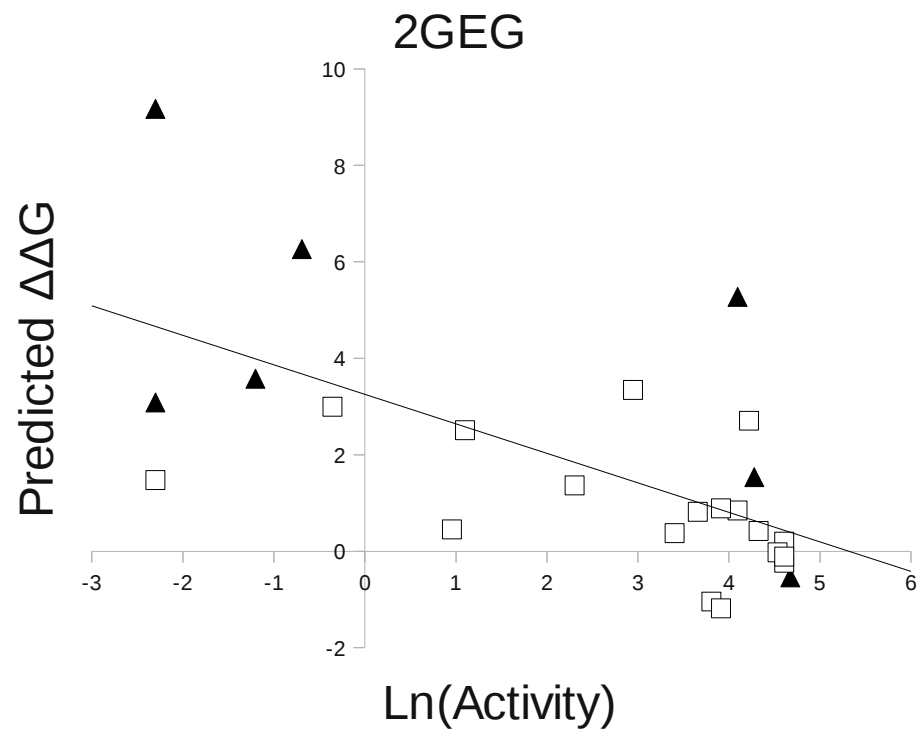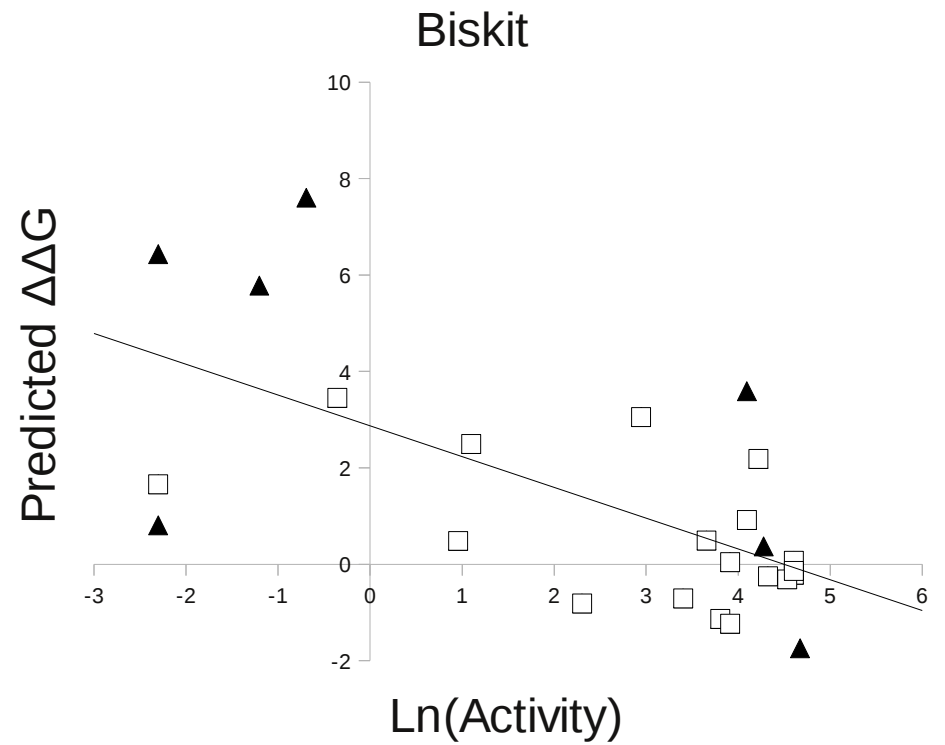

Supplement: Figure S2 — Correlation between experimental activities and predicted stabilities for both models. Residues known to impair protein function independently of protein stability were excluded. Mutants with similar predicted stabilities (within 1 kcal mol−1), and thus more reliable, are depicted in open squares while those with different values, are in black triangles. In solid black, a trend-line considering all depicted values. (PDF) [file pone.0015899.s004.pdf]

Figure S3

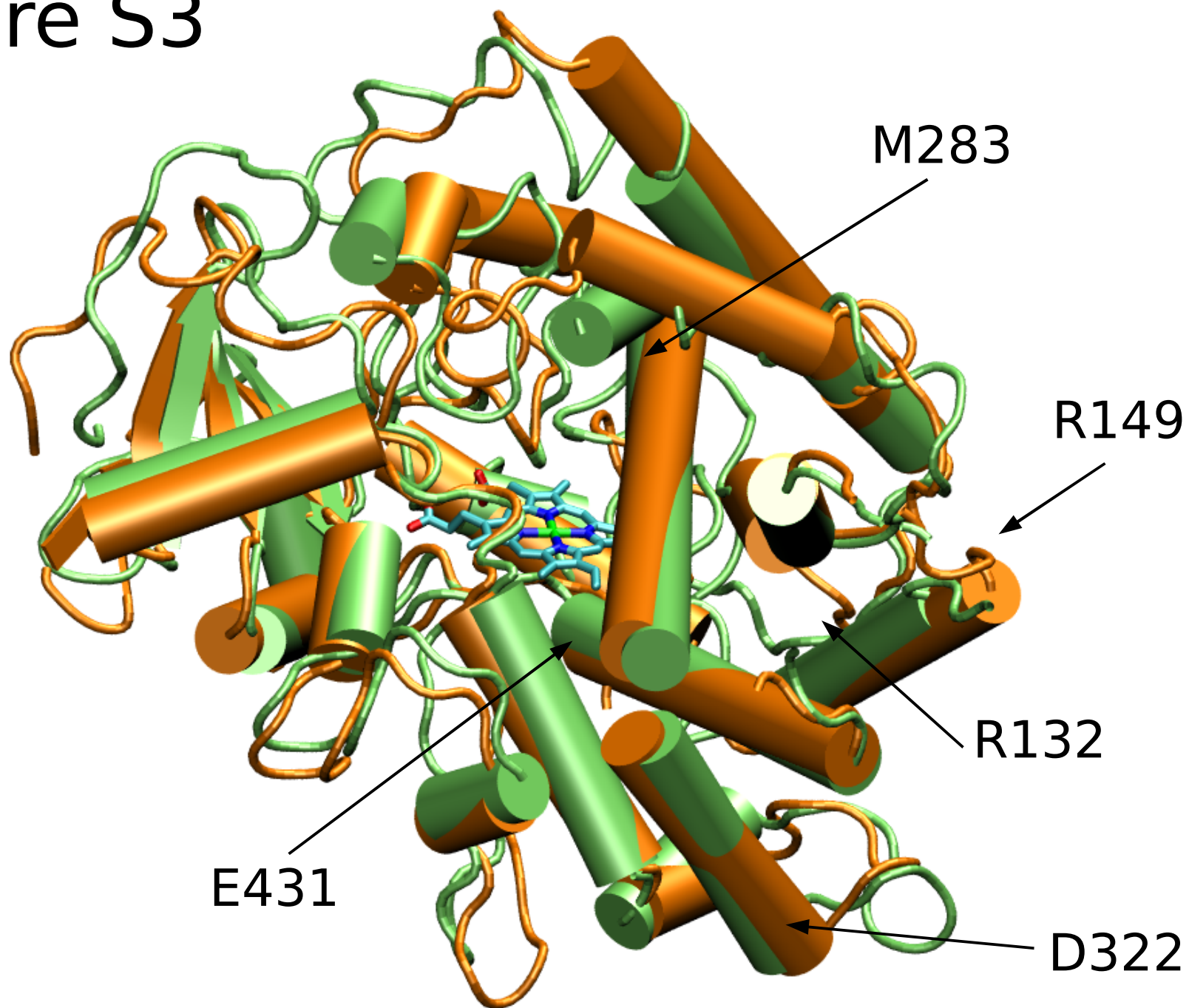

Supplement: Figure S3 — Superimposition of models 2GEG (orange) and Biskit (green). For clarity, only one heme is depicted in sticks. Both models tend to be more similar in regions with secondary structure. Residues implicated in the novel mutations found, as well as D322 residue are labeled (PDF) [file pone.0015899.s005.pdf]

# Figure S4

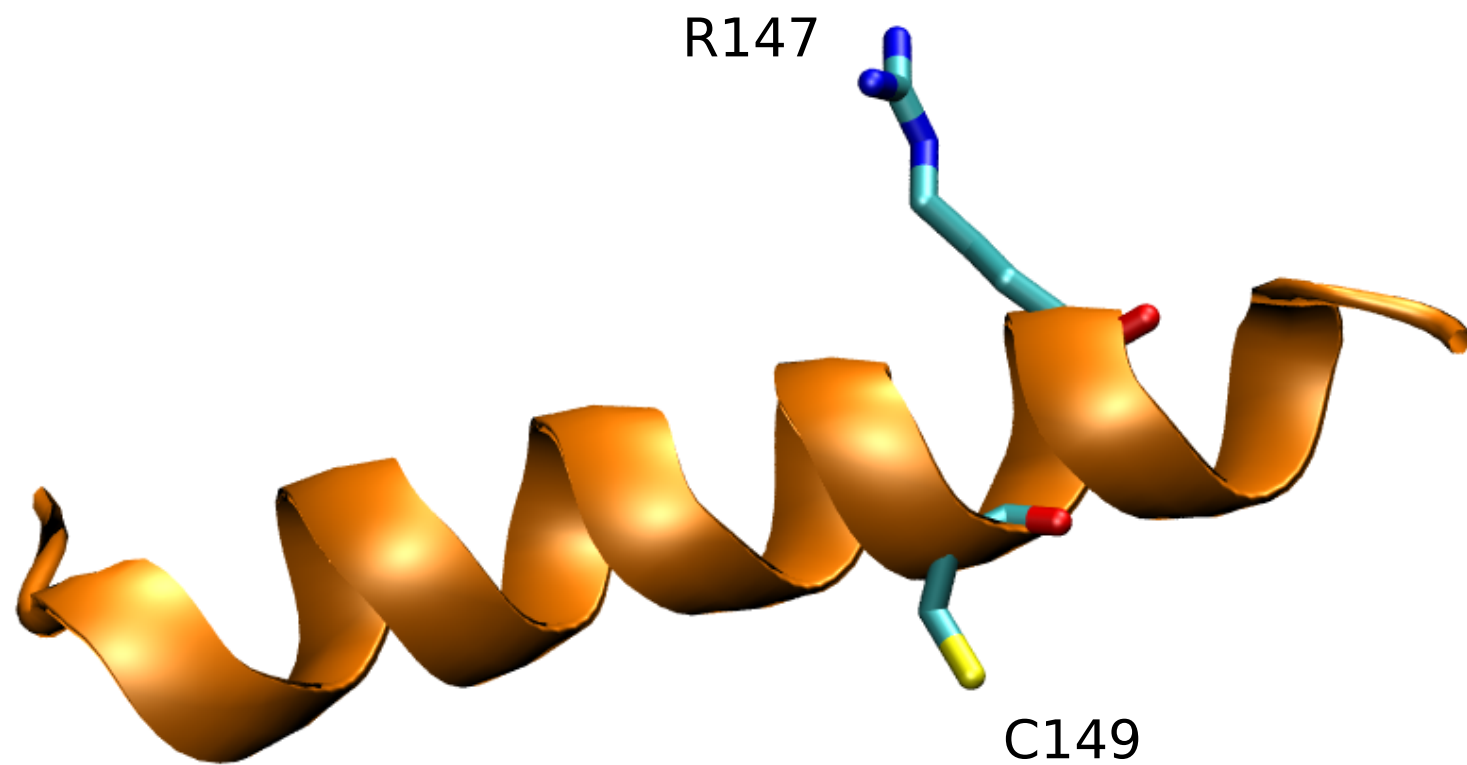

Supplement: Figure S4 — Cartoon representation of the helix D. Residues 147 and 149 (in sticks) point toward opposite directions. (PDF) [file pone.0015899.s006.pdf]
