# Supplementary material for: Structure-Based Analysis of Five Novel Disease-Causing Mutations in 21-Hydroxylase-Deficient Patients
Source: PLoS One. 2011 Jan 11;6(1):e15899. doi: 10.1371/journal.pone.0015899 (PMC3019215; doi:10.1371/journal.pone.0015899)
Supplement: Table S1 — Identity of the target sequence to the each template structure. PDB codes and chain identifier for the 18 template structures used along with their sequence identity to the aligned region. (DOC) [file pone.0015899.s007.doc]

| **Template structure** | **Sequence Identity**  **(%)** |
| --- | --- |
|  |  |
| 1CPT_A | 28 |
| 1IO7_ B | 30 |
| 1UE8_A | 30 |
| 1JFB_A | 22 |
| 1LFK_A | 24 |
| 1NR6_A | 31 |
| 1PQ2_B | 29 |
| 1R9O_A | 29 |
| 1PO5_A | 28 |
| 1TQN_A | 24 |
| 1X8V_A | 25 |
| 2CD8_B | 30 |
| 2F9Q_D | 31 |
| 2FDV_D | 27 |
| 2FR7_A | 30 |
| 2HI4_A | 28 |
| 2IJ2_B | 26 |
| 2OJD_B | 30 |

PDB structures where downloaded from the Protein Data Bank (<http://www.rcsb.org/>)
