# Supplementary material for: Structure-Based Analysis of Five Novel Disease-Causing Mutations in 21-Hydroxylase-Deficient Patients
Source: PLoS One. 2011 Jan 11;6(1):e15899. doi: 10.1371/journal.pone.0015899 (PMC3019215; doi:10.1371/journal.pone.0015899)
Supplement: Table S2 — Predicted ΔG and in vitro enzymatic activity of 40 point mutations of human P450CYP21A2. The mutations were analyzed in silico, by means of mutagenesis modeling and stability calculations using a previously available model and our own generated one, with the protein design algorithm FoldX. (DOC) [file pone.0015899.s008.doc]

|  | **Mutant** | **Predicted ΔG** | | **Measured**  **Activity (%)** | **Reference** |
| --- | --- | --- | --- | --- | --- |
|  |  | **2GEG** | **Biskit** |  |  |
|  | p.P30L | 0.85 | 0.92 | 60 | [1] |
| # | p.P30Q | 1.48 | 1.66 | 0 | [2] |
|  | p.I77T | 2.51 | 2.49 | 3 | [3] |
| * | p.G90V | 7.71 | 2.90 | 0 | [4] |
|  | p.P105L | 5.27 | 3.59 | 60 | [5] |
|  | p.L166P | 3.58 | 5.78 | 0.3 | [6] |
|  | p.I171N | 3.00 | 3.45 | 0.7 | [7] |
| * | p.I172N | 0.09 | 2.10 | 1 | [8] |
|  | p.G178A | 3.35 | 3.06 | 19 | [4] |
|  | p.D183E | -0.24 | 0.07 | 100 | [9] |
| # | p.I236N | 3.08 | 0.81 | 1 | [10] |
|  | p.S268C | -0.02 | -0.31 | 93 | [11] |
|  | p.S268M | -0.54 | -1.74 | 107 | [11] |
|  | p.S268T | -0.11 | -0.14 | 100 | [11] |
|  | p.V281I | -1.04 | -1.13 | 45 | [11] |
|  | p.V281L | -1.18 | -1.23 | 50 | [11] |
|  | p.V281T | 1.36 | -0.81 | 10 | [11] |
| * | p.G291S | 1.00 | -0.61 | 0.8 | [12] |
|  | p.R339H | 0.89 | 0.04 | 50 | [13] |
| * | p.R341P | 1.68 | 2.17 | 0.7 | [7] |
| * | p.E351D | 3.15 | 0.16 | 3.4 | [14] |
| * | p.E351I | 6.64 | 5.26 | 0.9 | [14] |
| * | p.E351K | 13.42 | 11.15 | 1.1 | [14] |
|  | p.R354H | 9.17 | 6.43 | 0 | [4] |
| * | p.R356P | 0.29 | 1.50 | 0.2 | [15] |
| * | p.R356Q | -1.26 | 0.25 | 1 | [15] |
| * | p.R356W | -0.63 | 1.34 | 0 | [16] |
|  | p.E380D | 0.38 | -0.71 | 30 | [17] |
|  | p.A391T | 0.82 | 0.49 | 38.7 | [6] |
| * | p.R426H | 0.66 | -0.37 | 0.5 | [7] |
| * | p.C428S | 0.26 | 0.84 | 0 | [11] |
| * | p.A434V | -0.08 | -1.32 | 14 | [3] |
|  | p.L446P | 6.26 | 7.60 | 0.5 | [7] |
|  | p.P453S | 2.71 | 2.18 | 68 | [5] |
|  | p.P463L | 0.45 | 0.48 | 2.6 | [18] |
|  | p.R479L | 0.42 | -0.25 | 75.5 | [6] |
|  |  | **Mutant** | **Predicted ΔG** | **Measured**  **Activity (%)** | **Reference** |
|  | p.P482S | 1.54 | 0.37 | 72 | [19] |
| * | p.R483P | 5.06 | -0.69 | 2 | [20] |
| * | p.R483Q | 1.85 | 0.25 | 1.1 | [6] |
|  | p.N493S | 0.2 | -0.22 | 100 | [21] |

***** residues involved in heme, ligand or protein binding; **#** residues excluded conditionally.

**Note**: Besides residues directly coordinating heme, we exclude the following:

Residue G90 is next to an Arginine that binds heme. As Glycine allows very flexible dihedral angles with neighboring residues, it is expected that p.G90V would impair heme binding (by R91) rather than the effect on stability itself. Residue E351 has been described to be part of ERR triad and its mutations is not linked to protein destabilization [16]. Residue R356 is surrounded by heme binding residues so we suggest that mutation by P or W residues would disrupt local conformation, thus impairing heme binding.

**REFERENCES:**

1. Tusie-Luna MT, Speiser PW, Dumic M, New MI, White PC (1991) A mutation (Pro30 to Leu) in CYP21 represents a potential nonclassic steroid 21-hydroxylase deficiency allele. Mol Endocrinol 5:685-692.
2. Lajic S, Nikoshkov A, Holst M, Wedell A (1999) Effects of missense mutations and deletions on membrane anchoring and enzyme function of human steroid 21-hydroxylase (P450c21). Biochem Bophys Res Commun 268: 14682-14686.
3. Krone N, Riepe FG, Grötzinger J, Partsch CJ, Sippell WG (2005) Functional characterization of two novel point mutations in the CYP21 gene causing simple virilizing forms of congenital adrenal hyperplasia due to 21-hydroxylase deficiency. J Clin Endocrinol Metab 90:445-454.
4. Nunez BS, Lobato MN, White PC, Meseguer A (1999) Functional análisis of four CYP21 mutations from Spanish patients with congenital adrenal hyperplasia. Biochem Biophys Res Commun 262: 635-637.
5. Nikoshkov A, Lajic S, Holst M, Wedell A, Luthman H (1997) Synergistic effect of partially inactivating mutations in steroid 21-hydroxylase deficiency. J Clin Endocrinol Metab 82:194-199.
6. Robins T, Bellanne-Chantelot C, Barbaro M, Cabrol S, Wedell A, et al. (2007) Characterization of novel missense mutations in CYP21 causing congenital adrenal hyperplasia. J Mol Med 85:247-255.
7. Barbaro M, Baldazzi L, Balsamo A, Lajic S, Robins T, et al. (2006) Functional studies of two novel and two rare mutations in the 21-hydroxylase gene. J Mol Med 84:521-528.
8. Tusie-Luna MT, Traktman P, White PC (1990) Determination of functional effects of mutations in the steroid 21-hydroxylase gene (CYP21) using recombinant vaccinia virus. J Biol Chem. 265:20916–20922.
9. Higashi Y, Hiromasa T, Tanae A, Miki T, Nakura J, et al. (1991) Effects of individual mutations in the P-450(C21) pseudogene on the P-450(C21) activity and their distribution in the patient genomes of congenital steroid 21-hydroxylase deficiency. J Biochem 109:638-644.
10. Robins T, [Barbaro M](http://www.ncbi.nlm.nih.gov/pubmed?term="Barbaro M"%5BAuthor%5D), [Lajic S](http://www.ncbi.nlm.nih.gov/pubmed?term="Lajic S"%5BAuthor%5D), [Wedell A](http://www.ncbi.nlm.nih.gov/pubmed?term="Wedell A"%5BAuthor%5D) (2005) Not all amino acid substitutions of the common cluster E6 mutation in CYP21 cause congenital adrenal hyperplasia. J Clin Endocrinol Metab 90:2148-2153.
11. Wu DA, Chung BC (1991) Mutations of P450c21 (steroid 21-hydroxylase) at Cys428, Val281, and Ser268 result in complete, partial, or no loss of enzymatic activity, respectively. J Clin Invest 88:519-523.
12. Wedell A, Ritzen EM, Haglund-Stengler B, Luthman H (1992) Steroid21-hydroxylase deficiency: three additional mutated alleles and establishment of phenotype-genotype relationships of common mutations. Proc Natl Acad Sci USA 89: 7232-7236.
13. Helmberg A, Tusie-Luna MT, Tabarelli M, Kofler R, White PC (1992) R339H and P453S: CYP21 mutations associated with nonclassic steroid 21-hydroxylase deficiency that are not apparent gene conversions. Mol Endocrinol 6: 1318-1322.
14. Krone N, Riepe FG, Grötzinger J, Partsch CJ, Brämswig J, et al. (2005) The residue E351 is essential for the activity of human 21-hydroxylase: evidence from a naturally occurring novel point mutation compared with artificial mutants generated by single amino acid substitutions. J Mol Med 83:561-568.
15. Lajic S, Levo A, Nikoshkov A, Lundberg Y, Partenen J, et al. (1997) A cluster of missense mutations at Arg356 of human steroid 21-hydroxylase may impair redox partner interactions. Hum Genet 99: 704-709.
16. Chiou SH, Hu MC, Chung BC (1990) A missense mutation at Ile172-Asn or Arg356-Trp causes steroid 21-hydroxylase deficiency. J Biol Chem 265:3549-3552.
17. Hsu NC, Guzov VM, Hsu LC, Chung BC (1999) Characterization of the consequence of a novel Glu-380 to Asp mutation by expression of functional P450c21in *Escherichia coli.* Biochim Biophys Acta 1430: 95-102.
18. Krone N, Riepe FG, Partsch CJ, Vorhoff W, Brämswig J, et al. (2006) Three novel point mutations of the CYP21 gene detected in classical forms of congenital adrenal hyperplasia due to 21-hydroxylase deficiency. Exp Clin Endocrinol Diabetes 114:111-117.
19. Barbaro M, Lajic S, Baldazzi L, Balsamo A, Pirazzoli P, et al. (2004) Functional analysis of two recurrent amino acid substitutions in the CYP21 gene from Italian patients with congenital adrenal hyperplasia. J Clin Endocrinol Metab 89:2402-2407.
20. Wedell A, Luthman H (1993) Steroid 21-hydroxilase (P450c21): a new allele and spread of mutations through the pseudogene. Hum Genet 91: 236-240.
21. Rodrigues NR, Dunham I, Yu CY, Carroll MC, Porter RR, et al. (1987) Molecular characterization of the HLA-lined steroid 21-hydroxylase B gene from an individual with congenital adrenal hyperplasia. EMBO J 6: 1653-1661.
